# Charcot Spine and Parkinson's Disease

**DOI:** 10.1155/2014/631346

**Published:** 2014-08-06

**Authors:** Philippe Loriaut, Sylvie Rozenberg, Patrick Boyer, Benjamin Dallaudière, Frederic Khiami, Elhadi Sariali, Hugues Pascal-Moussellard

**Affiliations:** ^1^Department of Orthopaedic Surgery, Pitié-Salpêtrière Hospital, Pierre and Marie Curie University, 47 Boulevard de l'Hôpital, 75013 Paris, France; ^2^Department of Rheumatology, Pitié-Salpêtrière Hospital, 47 Boulevard de l'Hôpital, 75013 Paris, France; ^3^Department of Orthopaedic Surgery, Bichat Claude Bernard Hospital, Paris Diderot University, 46 rue Henri Huchard, 75877 Paris, France; ^4^Department of Radiology, Bichat Claude Bernard Hospital, Paris Diderot University, 46 rue Henri Huchard, 75877 Paris, France

## Abstract

Charcot spine is rare condition whose association with Parkinson's disease (PD) has not been reported yet. The authors reported the cases of two patients with PD who developed Charcot spine. Both patients presented with a history of back pain and bilateral radicular leg pain. They had complete clinical and radiological assessment. Lumbar spine was involved in both patients. Clinical features and response to treatment were described. In the first case, circumferential fusion and stabilization were performed on the dislocated vertebral levels. A solid and stable fusion of the spine was obtained with satisfactory clinical outcome. Surgical treatment has been recommended to the other patient. In both cases, no other neurological etiology was found to account for Charcot spine. In conclusion, Charcot spine is associated with several neurological affections but has not previously been reported in association with Parkinson's disease.

## 1. Introduction

Charcot spine is a rare chronic disorder, characterized by progressive deformity, destruction, and severe instability of the spine. Repeated external forces associated with impaired defence mechanisms of the joints could be responsible for spinal injury. Various other terms have been used to describe this entity, including spinal neuroarthropathy, neuropathic spinal arthropathy, osteoarthropathy, neurotrophic joint, and neuroosteoarthropathy. This proliferation of terms probably reflects uncertainty about its pathogenesis [1].

Charcot first described neuropathic arthropathy occurring in tabetics patients and suggested that there was a causal relationship between spinal cord lesions and Charcot joints [2]. Kronig reported the first case of Charcot spine in a tabetic patient [3]. The thoracolumbar spine is the most common site affected and the lesion is often limited to one or two vertebrae [4]. Progressive deformation of the vertebral bodies leads to a kyphosis or kyphoscoliosis. Radiological features are quite characteristic [5]. Two types of neuropathic joints are described in the literature: hypertrophic and atrophic. The hypertrophic joint has massive new bone formation, osteophytes, osseous debris, and disruption of the articular surfaces. The atrophic form presents with osteopenia and osteolysis. The radiographic appearance is characterized by an extensive bone resorption without evidence of accompanying bone repair. Destruction coupled with massive bone formation may lead to subluxation of the vertebral body laterally, as well as disk spaces narrowing and ankylosis [1].

Many causes of neuropathic osteoarthropathy have been investigated including tabes dorsalis, spinal cord injuries, syringomyelia, leprosy, cord tumours, spinal tuberculosis, cerebrovascular accidents, diabetes, and alcoholic neuropathy (Table 1) [6–9]. To our knowledge, no association with Parkinson's disease (PD) has yet been reported.

The purpose of this case report was to describe the clinical and radiological features of two patients with Parkinson's disease presenting with neuropathic spinal arthropathy.

## 2. Case Report 1

A 72-year-old man was diagnosed 12 years ago as suffering from idiopathic PD. He has been taking dopaminergic treatment with a fair symptomatic control. His past medical history was significant for coronaropathy, pacemaker, and hemochromatosis. At the age of 64, the patient started to complain of increasingly invalidating, chronic back pain during movement of the lower lumbar region. This back pain was progressively associated with bilateral radicular leg pain and weakness. Clinical examination revealed motor function impairment at level L4/L5 with a leg motor scale score of 3 out of 5. He first presented at an orthopaedic surgery department where he had undergone L3–L5 laminectomies for degenerative lumbar stenosis. Surgery had only a limited and temporary benefit. Indeed, the patient reported six months postoperatively low back and right radicular leg pain recurrence with foot drop. Motor function remained impaired with a marked hypoesthesia along L4 dermatome. Electromyography revealed right L4, L5, and S1 irritation.

Medical treatment and epidural cortisone injections remained unsuccessful over the years. Lumbocrural pain and neurological impairment were progressively associated with thoracolumbar kyphosis.

**Figure 1:**
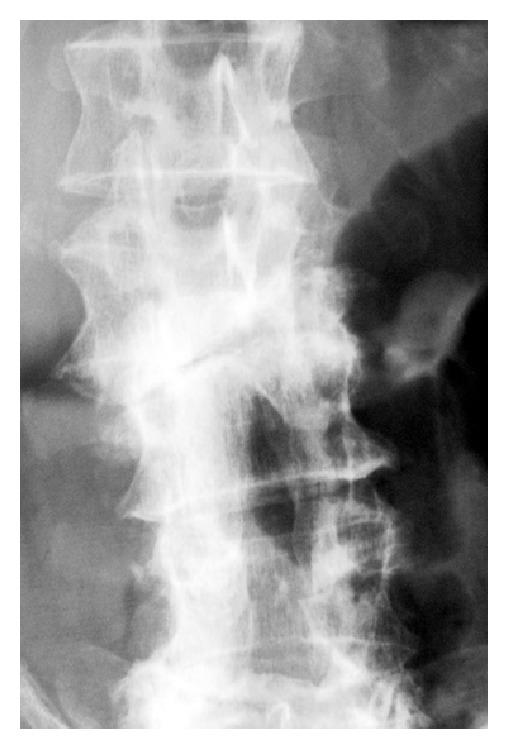
Anteroposterior radiographs showing marked narrowing of L2-L3 disc space with some endplate destruction associated with new bone formation and a severe lateral dislocation of the L2/L3 joint (Case 1).

**Figure 2:**
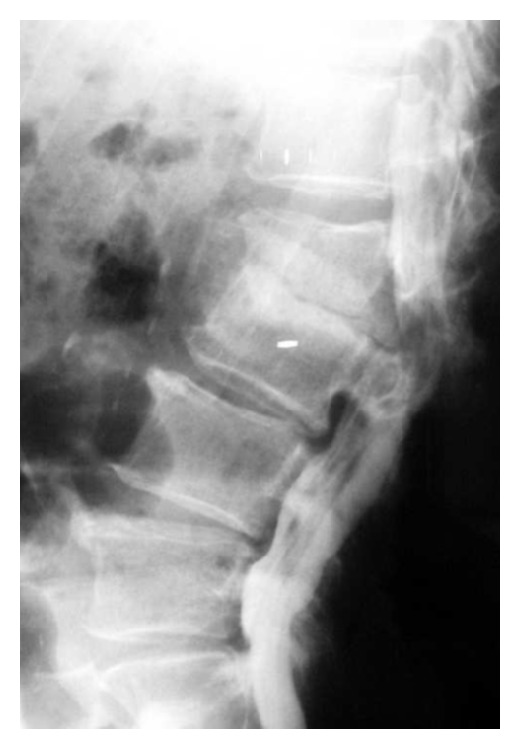
Preoperative plain radiograph (lateral view) showing marked narrowing of L2-L3 disc space with some endplate destruction; new bone formation is noted (Case 1).

**Figure 3:**
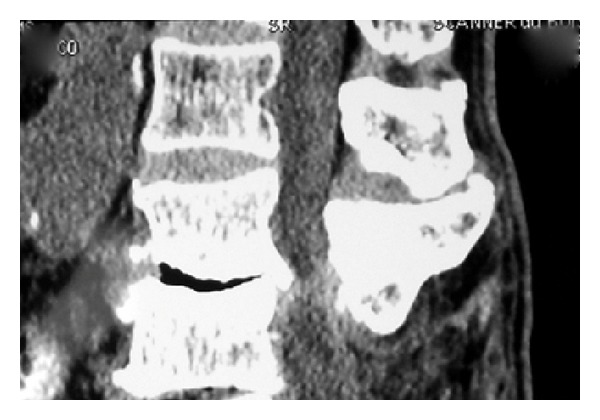
CT scan showing a destruction of L2 vertebral body and L2-L3 disc space. The spinal canal was compromised with paravertebral hypertrophic ossifications (Case 1).

Three years postoperatively, the patient was referred to our institution. Extensive radiological assessment was realized with analysis of previous imaging compared to new ones. Plain radiographs revealed a combination of an extensive discovertebral destruction with L2 degenerative collapse associated with paravertebral hypertrophic ossifications. Lesions developed at the upper limit of the laminectomized area (Figures 1 and 2). Dynamic X-ray imaging revealed the extent of the spinal instability. CT scan showed a severe lateral dislocation of the L2/L3 joint associated with a reduced local kyphosis angle (Figure 3).

The progressive onset of associated destructive and constructive radiological phenomena provided arguments for an osteoarthropathy and raised suspicions of Charcot spine. Firstly, investigations focused on differential diagnosis and etiological research. Blood tests and cultures including serologic test for syphilis were performed. There was no evidence of infection or an inflammatory syndrome. Nevertheless, the hypotheses of a chronic vertebral osteomyelitis or a tumoral process were explored. A CT-guided vertebral biopsy was consequently performed. Cultures proved negative. Histological examination revealed the presence of fibrous tissue with sequestered bone but no signs of malignancy. Screening for the HLA B27 gene was negative. Investigations concluded that the patient had a neurological osteoarthropathy with an isolated PD.

**Figure 4:**
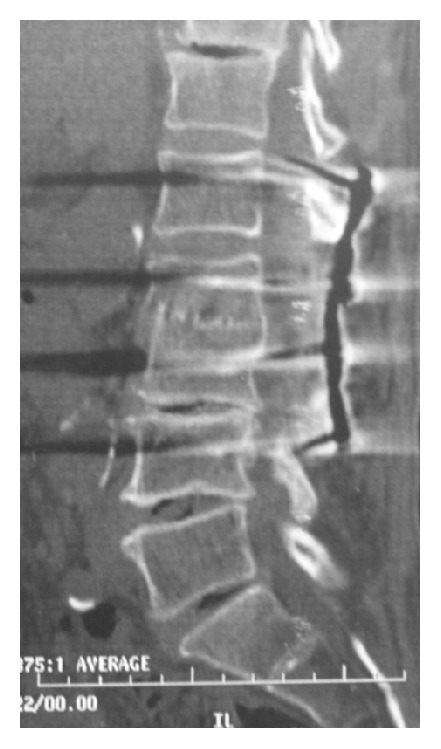
CT scan at the 5-year followup confirming bony union achievement and a solid and stable fusion of the spine (Case 1).

Further surgery was decided in order to decompress and stabilize the spine. The patient underwent decompression and transforaminal lumbar interbody fusion (TLIF). First laminectomy at L3 was performed; then, each L3/L4 nerve root was decompressed. Foraminotomy and bilateral facetectomy provided access to the L2/L3 intervertebral disc. A vacuum disc phenomenon was noted at this level. Discectomy was thus reduced to a minimum. Finally, disc space was replaced by intersomatic bone graft. The fusion from L1 to L4 was performed using pedicle screws, rods, and a posterolateral bone graft. The patient was placed in a thoracolumbosacral orthosis during 3 months. Afterwards, the patient's condition improved progressively. Three months postoperatively, he had a significant pain relief and complete weakness recovery. At the 5-year follow-up examination, clinical outcome was satisfactory and radiological assessment confirmed that the bony union was achieved and that a solid and stable fusion of the spine was obtained (Figure 4).

## 3. Case Report 2

**Figure 5:**
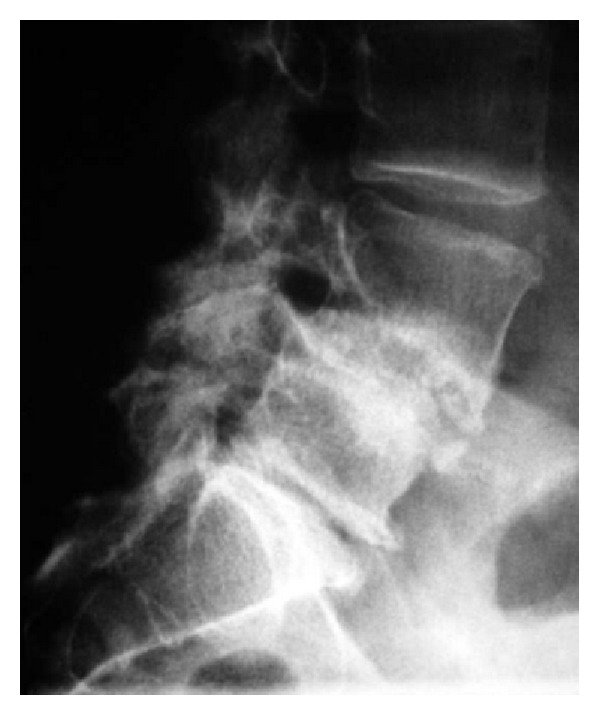
Lateral radiograph shows destruction of L4-L5 vertebral bodies producing multiple bony fragments (Case 2).

**Figure 6:**
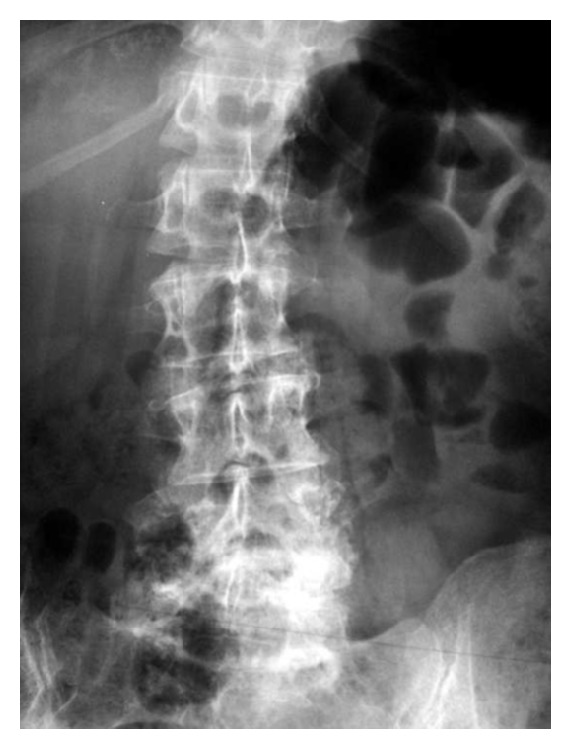
Anteroposterior radiograph shows leftward translation of L4 (Case 2).

**Figure 7:**
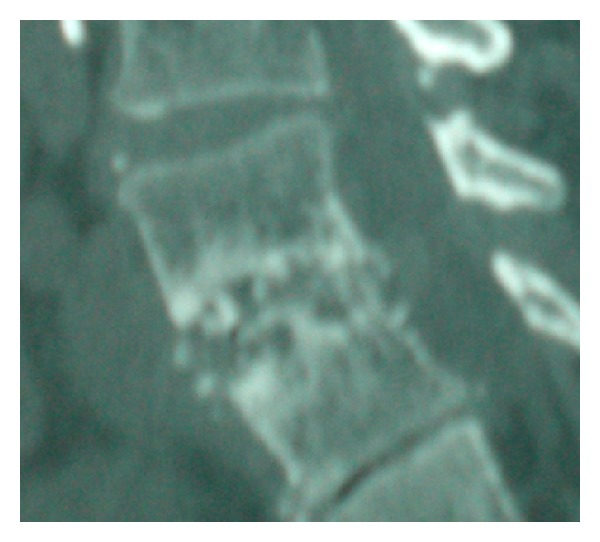
Sagittal CT scan image showing bony destruction of L4 and L5 (Case 2).

A 62-year-old woman was diagnosed with PD about eight years ago. She received dopaminergic treatment and underwent deep brain stimulation five years ago which provided a significant improvement in her symptoms. Four years ago, she was referred to our department of rheumatology for a 2-year history of low back pain radiating down her buttock, the lateral side of the thigh, and the leg. Symptoms were predominant on the right side and were not responsive to a conservative analgesic treatment. The range of motion was quite limited. She had to stop her professional activity a few months after the onset since pain kept increasing. Physical examination revealed mild tenderness of the lower back, a positive Lasègue sign on the right leg. Jerks were active on both sides. Neither motor weakness nor hypoesthesia was detected. Comparing radiographs and CT scan images showed a progressive onset of destruction images of L4-L5 vertebral bodies associated with production of multiple bony fragments and leftward translation of L4 (Figures 5, 6, and 7). Those elements led to the hypothesis of a Charcot spine. The same work-up as in case report 1 was thus conducted. Histological examination showed nonspecific bone necrosis without evidence of chronic vertebral infection or tumoral process. The patient received epidural transforaminal L4-L5 steroid injection under radioscopic guidance. Initially, she experienced a significant pain relief but symptoms recurred two months later. The patient was offered a surgical treatment but declined it. Symptomatic treatment was therefore pursued.

## 4. Discussion

A great polymorphism of initial clinical features of this disease is depicted in the literature [1, 4, 8, 10–12]. Most patients initially presented with very common and unspecific symptoms, such as progressive back and leg pain and weakness. Besides, it turns out that patients reported a rather insidious onset and tolerated their symptoms for several years before seeking medical attention leading to a delay in diagnosis. Furthermore, these presentations may belong to a large panel of pathologies, making it difficult for the clinician to first suspect a neurological osteoarthropathic lesion.

In our study, both patients initially demonstrated particular clinical features, supporting first—and incorrect—diagnosis of degenerative lumbar stenosis. Imaging might also have been confusing as it depicted some features compatible with a degenerative etiology. This presentation was responsible for a misguided work-up and therapeutic management.

It should be noted that presentation in the first case probably differed from the second one due to surgical management. Later on, both patients presented more typical radiological images providing arguments for a neurological-related osteoarthropathy. Diagnosis was only reconsidered after, respectively, 8 and 4 years after the onset of symptoms.

There are two main theories describing the pathogenesis of neuropathic osteoarthropathy. The neurotraumatic theory holds that loss of sensation and repetitive trauma may be primary pathogenetic mechanisms for Charcot joint destruction [13, 14]. It suggests that unrecognized trauma results in occult bone and joint injuries. In patients with sensory peripheral neuropathy, these injuries remain undetected, leading to hyperaemia, soft tissue swelling, and bone alteration. On the other hand, according to the neurovascular theory, an underlying vascular abnormality could be responsible for bone destruction as described in diabetes [1, 15].

Recently, other investigators have suggested that Charcot's osteoarthropathy could involve abnormal bone metabolism and neuroregulation [16]. Several neural transmission ligands have been demonstrated to be in close spatial association with osteoblasts and osteoclasts. Further, receptors for these neural ligands are expressed on bone cells and administration of these neural transmission molecules has potent effects on bone cells. Neurotransmission impairment could thus be responsible for bone metabolism alteration in disorders of the central and peripheral nervous system.

Several reports have been published documenting the existence of this uncommon clinical entity [10–12] but this appears to be the first two cases associated with PD recorded in the literature. PD is one of the most common neurodegenerative diseases. It is characterized by clinical manifestations: stiffness, postural instability, and insensate vertebral joint microtraumatisms caused by movement disorders. Physiopathological features include altered bone metabolism and defective neuroosseous signal transmission.

This case report is also consistent with studies that emphasize that spine surgery in patients with PD is complex and may lead to several complications [17].

In conclusion, Charcot spine is associated with several neurological affections but has not previously been reported in association with Parkinson's disease. As its physiopathology is still unknown, this case report emphasizes that there could be a possible association between both conditions. However, we are not able to exclude the possibility of a simple coincidental phenomenon. Further experimental and clinical investigations will be necessary for a better understanding of nerve-bone interaction.

## Figures and Tables

**Table 1:** Diseases associated with Charcot's osteoarthropathy.

Syphilis (tabes dorsalis)	
Leprosy	
Diabetes	
Spina bifida	
Congenital insensitivity to pain	
Syringomyelia	
Cerebral palsy	
Spinal cord injury	
Spinal cord compression	
Peripheral nerve injuries	
Alcoholism	
Amyloidosis	
Amyotrophic lateral sclerosis	
Arachnoiditis	
Cerebrovascular accident	
Cord trauma/tumors	
Elephantiasis	
Hereditary insensitivity to pain	
Hereditary sensory radicular neuropathy	
Lead poisoning	
Meningomyelocele	
Multiple sclerosis	
Pernicious anemia	
Yaws	
